# Real-time tropospheric delay retrieval with GPS, GLONASS, Galileo and BDS data

**DOI:** 10.1038/s41598-018-35155-3

**Published:** 2018-11-20

**Authors:** Lin Pan, Fei Guo

**Affiliations:** School of Geodesy and Geomatics, Wuhan University, 129 Luoyu Road, Wuhan, 430079 China

## Abstract

The precise point positioning (PPP) is a promising technology for the real-time retrieval of atmospheric parameters with a single receiver in anywhere, all-weather and any time. The real-time atmospheric parameters can be applied to the time-critical meteorology, such as the severe weather nowcasting. The PPP is a satellite-based technology. Multi-constellation integration can enhance satellite geometry and increase measurement redundancy so that the solutions of atmospheric parameters are expected to be improved. Currently, the Global Navigation Satellite System (GNSS) family includes recovered GLONASS and modernized GPS as well as the emerging Galileo and BDS. A week of GNSS observations from 160 stations are processed to retrieve the tropospheric zenith total delay (ZTD) in real time. The four-constellation mixed real-time precise products including satellite orbit and clock corrections are adopted, and their quality is evaluated. The performance of ZTD estimates is assessed in terms of accuracy and convergence time by comparing with final tropospheric ZTD products provided by two analysis centers. The ZTDs retrieved from different constellation combinations (i.e., GPS/GLONASS/Galileo/BDS, GPS/GLONASS, and GPS-only), different processing models for ionospheric delays (i.e., ionospheric-free (IF) combined PPP, and uncombined (UC) PPP), and different modes (i.e., real-time mode, and post-processing mode) are compared.

## Introduction

During various processes of atmosphere, such as climate changes, hydrological cycle, and atmospheric radiation, an important role is played by the atmospheric water vapor. The meteorological sensors are first used to measure the atmospheric water vapor, including water vapor radiometer and radiosonde. However, there are some disadvantages for the traditional measurement techniques. The low spatiotemporal resolution and high cost limit their applications. The GPS meteorology with the use of ground-based receivers for sounding water vapor was first introduced in the early 1990s^1^. Due to its all-weather capability, broad spatial coverage, low operational expense, and high temporal resolution, many efforts have been made to retrieve atmospheric water vapor using GPS double-difference (DD) code and carrier phase observations since then^2–4^. The results indicated that the accuracy of GPS-based water vapor estimates is comparable to that derived from meteorological sensors^5^. In the relative positioning strategy, the simultaneous observations should be formed and the proper baselines should be selected. Therefore, the data processing of this strategy is relatively complex, especially for the increasingly dense ground tracking networks used for regional applications. The precise point positioning (PPP) technique, with the use of the precise satellite orbit and clock products and the observations from a single receiver, can obtain the absolute zenith total delays (ZTDs)^6^, while only the differences between ZTDs of two different stations can be acquired for the relative positioning approach. As the PPP outperforms the relative positioning in terms of observation requirements and computational efficiency, it has been a promising technique for GPS meteorology.

The numerical weather prediction (NWP) models continuously assimilate the near-real-time tropospheric products generated with GPS measurements including precipitable water vapor (PWV) and ZTDs^7,8^. The benefits of tropospheric products for the forecasting of precipitation and cloud were demonstrated^9,10^. Yao *et al*.^11^ proposed a short-term rainfall forecasting method by studying the relationship between time-varying PPP-derived PWV and rainfall in the post-processing mode. The results indicated that the forecasted correct rate could reach approximately 80%, while the false alarm rate was approximately 66%. Despite the applications of tropospheric products, most of the previous studies were confined to post-processing or near-real-time modes. For some innovative applications, such as severe weather nowcasting, the information of atmospheric state should be updated with very short or no latency, since the atmospheric water vapor has fast spatiotemporal variability^12^. For the community of GPS meteorology, a focus is the real-time delivery of tropospheric products to serve time-critical operational meteorology. Real-time retrieval of ZTDs or PWV using PPP technique needs the application of real-time precise corrections, including satellite orbits and clocks. The International Global Navigation Satellite System (GNSS) Service (IGS) Real-Time Pilot Project (RTPP) commits to the generation and distribution of real-time precise satellite orbit and clock products. Due to recent IGS RTPP development, these products are currently available for scientific studies, which provides a good potential for the real-time ZTD estimation with the use of PPP. Several researchers focused on the real-time ZTD or PWV retrieval with GPS-only PPP^13,14^. The results indicated that an accuracy of several millimeters could be achieved when taking the meteorological data or post-processing products as references. Zhao *et al*.^15^ first analyzed the time series of GPS-only real-time PPP-derived ZTD by a least-square fitting of the broken line tendency for the observations spanning a whole year, and then, proposed a nowcasting method for precipitation based on the ZTD slope in the ascending period. Numerical results showed that the proposed method could predict approximately 85% of the precipitation events in the year from September 1, 2014 to August 31, 2015.

A rapid development has been undergone for the world of satellite systems in recent years. The GNSS family is extended from a single GPS constellation to four constellations with Galileo, BDS, GLONASS and GPS. As of February 2018, there are 22 Galileo satellites, 26 BDS satellites, 24 GLONASS satellites, and 31 GPS satellites in operation. When all four constellations are fully deployed, the available GNSS satellites will be up to more than 120. The PPP is a satellite-based technology, and its performance is mainly determined by the tracked satellites^16,17^. Attributing to the enhanced geometry and increased number of visible satellites, multi-constellation integration is anticipated to improve the availability, reliability, stability, and accuracy of PPP-derived ZTD or PWV products in comparison to a single constellation^18^. Ding *et al*.^19^ investigated the real-time estimation of tropospheric delays based on multi-GNSS PPP. The observations from only three satellite systems were employed, and the emerging new satellite system BDS was not included in their analysis. Lu *et al*.^20^ carried out the investigation on real-time ZTD retrieval with four-constellation integrated PPP. The improvement of ZTD estimates for four-constellation integrated PPP solutions on initialization time and accuracy was 8% and 22% compared with GPS-only PPP solutions, respectively. However, the tropospheric azimuthal asymmetry was ignored in their results. In view that the position solutions with improved accuracy could be achieved if high-resolution horizontal atmospheric gradients were estimated^21^, the consideration of horizontal atmospheric distribution is also expected to enhance the sensing of ZTDs. In addition, the effects of different handling strategies of ionospheric delays on the real-time ZTD estimation are still unclear. The ionospheric-free (IF) combined model^14,15,18,19^ was mostly adopted by previous studies in the PPP processing, while few studies related to the uncombined (UC) model.

Overall, most of the previous studies were confined to the processing mode (post-processing mode), data for ZTD retrieval (GPS-only or GPS/GLONASS based PPP), modeling of the tropospheric delays (ignoring the tropospheric azimuthal asymmetry), or the handing strategies of ionospheric delays (IF combined model). This study performs a comprehensive consideration of the above four aspects so as to derive the high-accuracy real-time ZTDs. The superiority of our work can be summarized as: this study focuses on the real-time retrieval of tropospheric ZTDs using four-constellation integrated PPP with Galileo, BDS, GLONASS and GPS measurements; we carefully consider the horizontal distribution of atmosphere by introducing atmospheric gradients; we compare the ZTDs derived from the IF combined PPP and UC PPP. The performance of real-time ZTD estimates is evaluated in terms of the convergence time and the accuracy with respect to the high-accuracy post-processed ZTD products provided by two IGS analysis centers. The contribution starts with the ZTD estimation approach with multi-GNSS PPP in real time. Subsequently, the datasets and products are introduced, including multi-GNSS observations, real-time precise satellite orbit and clock products, and references of tropospheric ZTD estimates. Next, we present our results in terms of real-time ZTDs derived from IF combined PPP, effects of tropospheric asymmetry on real-time ZTD retrieval, real-time ZTDs in constrained visibility environments, real-time ZTDs derived from UC PPP, and post-processed ZTDs derived from IF combined PPP. Finally, the conclusion and discussion are summarized.

## Ztd Estimation With Multi-Gnss Ppp In Real Time

The code and carrier phase observations on a single frequency can be shown as follows:

1 $$\{\begin{array}{rcl}{p}_{r,j}^{S} & = & {\rho }_{r}^{S}+d{t}_{r}-d{t}^{S}+{I}_{r,1}^{S}\cdot {\gamma }_{j}+{T}_{r}^{S}+{\varepsilon }_{r,j}^{S}+{\delta }_{r,j}^{S}\\ {\varphi }_{r,j}^{S} & = & {\rho }_{r}^{S}+d{t}_{r}-d{t}^{S}-{I}_{r,1}^{S}\cdot {\gamma }_{j}+{T}_{r}^{S}+{N}_{r,j}^{S}+{\xi }_{r,j}^{S}+{d}_{r,j}^{S}\end{array}$$

with ${\gamma }_{j}={f}_{1}^{2}/{f}_{j}^{2}$ and $j=(1,\,2)$ where *S* represents the GNSS satellite, *r* represents the receiver, *p* and *ϕ* denote the measured code and carrier phase, respectively, *ρ* denotes the geometric distance between the receiver and the satellite, *dt*_*r*_ denotes the receiver clock offsets, *dt*^*S*^ denotes the satellite clock offsets, *I*_1_ denotes the slant ionospheric delays on L1/G1/B1/E1 frequencies, ƒ denotes the carrier frequency, *T* denotes the slant tropospheric delays, *N* denotes the phase ambiguities, *ε* and *ξ* refer to the sum of multipath errors and measurement noises for code and phase observables, respectively, and *δ* and *d* denote the code and phase hardware delays, respectively. In order to derive the high-accuracy estimates for the tropospheric delays, the other terms in Equation (1) should be corrected with precise products and established models, or properly estimated as unknown parameters. It is important to notice that some terms are not estimable for a single station due to the rank deficiency, such as the satellite orbits and clocks. In the standard PPP processing, the satellite orbits and clocks are first determined by the analysis centers with the network solutions based on hundreds of ground tracking stations, and then, broadcast to the PPP users. In this way, the precise satellite orbits and clocks can ensure the estimation accuracy of other terms, such as tropospheric delays and receiver positions. Although the receiver positions can be estimated together with the tropospheric delays, the performance of the estimates for the later ones will degrade^20^. All the stations used in this study are IGS stations, and their coordinate values with an accuracy of several millimeters are weekly released by the IGS. To pursue the accurate tropospheric delay estimates, the IGS solutions are adopted for the receiver coordinates.

After applying the real-time precise corrections of satellite orbits and clocks and fixing the station coordinates, the multi-GNSS PPP observation model based on UC observations can be written as:

2 $$\{\begin{array}{rcl}{P}_{r,j}^{G} & = & d{t}_{r}+{I}_{r,1}^{G}\cdot {\gamma }_{j,G}+{T}_{r}^{G}+{\varepsilon }_{r,j}^{G}\\ {P}_{r,j}^{R} & = & d{t}_{r}+{I}_{r,1}^{R}\cdot {\gamma }_{j,{R}_{k}}+{T}_{r}^{R}+{\varepsilon }_{r,j}^{R}+{b}_{r,{R}_{k},G}\\ {P}_{r,j}^{C} & = & d{t}_{r}+{I}_{r,1}^{C}\cdot {\gamma }_{j,C}+{T}_{r}^{C}+{\varepsilon }_{r,j}^{C}+{b}_{r,C,G}\\ {P}_{r,j}^{E} & = & d{t}_{r}+{I}_{r,1}^{E}\cdot {\gamma }_{j,E}+{T}_{r}^{E}+{\varepsilon }_{r,j}^{E}+{b}_{r,E,G}\end{array}$$

3 $$\{\begin{array}{rcl}{{\rm{\Phi }}}_{r,j}^{G} & = & d{t}_{r}-{I}_{r,1}^{G}\cdot {\gamma }_{j,G}+{T}_{r}^{G}+{N}_{r,j}^{G}+{\xi }_{r,j}^{G}\\ {{\rm{\Phi }}}_{r,j}^{R} & = & d{t}_{r}-{I}_{r,1}^{R}\cdot {\gamma }_{j,{R}_{k}}+{T}_{r}^{R}+{N}_{r,j}^{R}+{\xi }_{r,j}^{R}\\ {{\rm{\Phi }}}_{r,j}^{C} & = & d{t}_{r}-{I}_{r,1}^{C}\cdot {\gamma }_{j,C}+{T}_{r}^{C}+{N}_{r,j}^{C}+{\xi }_{r,j}^{C}\\ {{\rm{\Phi }}}_{r,j}^{E} & = & d{t}_{r}-{I}_{r,1}^{E}\cdot {\gamma }_{j,E}+{T}_{r}^{E}+{N}_{r,j}^{E}+{\xi }_{r,j}^{E}\end{array}$$

where *E*, *C*, *R*, and *G* represent the Galileo, BDS, GLONASS and GPS satellites, respectively, *k* denotes the frequency factor of GLONASS satellites, *P* denotes the OMC (observed minus computed) code observables, Φ denotes the OMC phase observables, and *b*_*r*_ denotes the inter-system bias (ISB) or inter-frequency bias (IFB). The receiver clocks are linearly correlated with receiver-dependent code hardware delays, and thus they are usually estimated as a lumped term in PPP. Both the signal structure and frequency are different for different satellite systems. As a result, the code hardware delays at the receiver within a multi-GNSS receiver differ among the four satellite systems. To solve this issue, a receiver clock offset parameter should be designed for each satellite system. Alternatively, the ISB can be introduced to compensate the difference between receiver clock estimates of different satellite systems. We choose the GPS system as the reference. A common ISB parameter is added to the code observations on two frequencies of all Galileo satellites, and it is the same case for BDS. In view that the frequency division multiple access (FDMA) technique is adopted by the GLONASS, the receiver code hardware delays of GLONASS are satellite dependent. Therefore, a respective IFB parameter should be estimated for each GLONASS frequency. As to the satellite-dependent code hardware delays, they are removed when correcting the precise satellite clocks, and no additional consideration is required. It is important to note that the phase ambiguities in Equation (3) assimilate the phase hardware delays.

For the purpose of strengthening the solutions, the temporal correlation of ionospheric delays is used to constrain their estimates, that is:

4 $${I}_{r,1}^{S}(t)-{I}_{r,1}^{S}(t-1)=w(t),\,w(t) \sim N(0,{\sigma }_{w}^{2}(t))$$

where *t* and *t* − 1 refer to two adjacent epochs, and *w*(*t*) is a zero mean white noise with a variance ${\sigma }_{w}^{2}(t)$. In this study, the variance is set to 1 × 10^−4^ m^2^ for the observations with a sampling rate of 30 s, and the elevation-dependent weighting scheme is adopted.

The slant tropospheric delays can be modeled by a sum of hydrostatic and wet components as well as the tropospheric gradients when the homogeneity and inhomogeneity of troposphere are considered, that is:

5 $${T}_{r}^{S}={{\rm{Mh}}}_{r}^{S}\cdot {{\rm{Zh}}}_{r}+{{\rm{Mw}}}_{r}^{S}\cdot {{\rm{Zw}}}_{r}+{{\rm{Mg}}}_{r}^{S}\cdot [{G}_{r,N}\cdot \,\cos (a)+{G}_{r,E}\cdot \,\sin (a)]$$

where Mh and Mw denote the hydrostatic and wet mapping functions, respectively, which can be both obtained using the Global Mapping Function (GMF)^22^, Mg denotes the mapping functions of gradients^23^, Zh_r_ denotes the zenith hydrostatic delays (ZHDs), which can be accurately calculated with the Saastamoinen model^24^ based on the pressure derived from the Global Pressure and Temperature 2 (GPT2) model^25^, Zw_*r*_ denotes the zenith wet delays (ZWDs), *G*_*r*,*E*_ and *G*_*r*,*N*_ denote the tropospheric horizontal gradients in east and north directions, respectively, and *a* denotes the satellite azimuth angles. The two gradient components and ZWDs are usually estimated as unknown parameters in PPP processing, since they cannot be corrected well. In the traditional modeling of slant tropospheric delays, the two gradient components shown in Equation (5) are absent due to the negligence of tropospheric azimuthal asymmetry.

All the unknown parameters are estimated by simultaneously processing the observations from all four satellite systems in a common estimator for the real-time PPP. For the atmospheric parameter retrieval using the UC multi-GNSS PPP, the unknown parameters include the ZWD, north-south gradient component, east-west gradient component, receiver clocks, ionospheric delays, float phase ambiguities, GPS–GLONASS IFB, GPS–BDS ISB, and GPS–Galileo ISB. The number is 1, 1, 1, 1, *n*_*S*_, 2 × *n*_*S*_, *n*_*k*_, 1, and 1 for the above nine kinds of parameters at a station, respectively, assuming that the number of visible GNSS satellites and GLONASS frequencies is *n*_*s*_ and *n*_*k*_, respectively. The unknown parameters can be depicted as:

6 $${X}_{UC}={[{{\rm{Zw}}}_{r},{G}_{r,N},{G}_{r,E},d{t}_{r},{I}_{r,1}^{S},{N}_{r,j}^{S},{b}_{r,{R}_{k},G},{b}_{r,C,G},{b}_{r,E,G}]}^{{\rm{T}}}$$

where *X*_*UC*_ is the vector of estimates.

As the ionospheric delay has dispersive nature, the IF combinations can be formed to eliminate its first-order effects, so that the complicated handing of ionospheric delay can be avoided. For comparison, the four-constellation integrated PPP based on IF combined observations is also used to estimate the ZTD in real time. The IF combined multi-GNSS PPP observation model can be expressed as:

7 $$\{\begin{array}{rcl}{P}_{r,IF}^{G} & = & d{t}_{r}+{T}_{r}^{G}+{\varepsilon }_{r,IF}^{G}\\ {P}_{r,IF}^{R} & = & d{t}_{r}+{T}_{r}^{R}+{\varepsilon }_{r,IF}^{R}+{b}_{r,{R}_{k},G}\\ {P}_{r,IF}^{C} & = & d{t}_{r}+{T}_{r}^{C}+{\varepsilon }_{r,IF}^{C}+{b}_{r,C,G}\\ {P}_{r,IF}^{E} & = & d{t}_{r}+{T}_{r}^{E}+{\varepsilon }_{r,IF}^{E}+{b}_{r,E,G}\end{array}$$

8 $$\{\begin{array}{rcl}{{\rm{\Phi }}}_{r,IF}^{G} & = & d{t}_{r}+{T}_{r}^{G}+{N}_{r,IF}^{G}+{\xi }_{r,IF}^{G}\\ {{\rm{\Phi }}}_{r,IF}^{R} & = & d{t}_{r}+{T}_{r}^{R}+{N}_{r,IF}^{R}+{\xi }_{r,IF}^{R}\\ {{\rm{\Phi }}}_{r,IF}^{C} & = & d{t}_{r}+{T}_{r}^{C}+{N}_{r,IF}^{C}+{\xi }_{r,IF}^{C}\\ {{\rm{\Phi }}}_{r,IF}^{E} & = & d{t}_{r}+{T}_{r}^{E}+{N}_{r,IF}^{E}+{\xi }_{r,IF}^{E}\end{array}$$

where *P*_*IF*_ and Φ_*IF*_ denote the IF combined OMC code and phase observables, respectively. The corresponding estimates vector can be described as follows:

9 $${X}_{IF}={[{{\rm{Zw}}}_{r},{G}_{r,N},{G}_{r,E},d{t}_{r},{N}_{r,IF}^{S},{b}_{r,{R}_{k},G},{b}_{r,C,G},{b}_{r,E,G}]}^{{\rm{T}}}$$

Partial estimated parameters of IF combined PPP are different from those of UC PPP, including the float phase ambiguities equal to the number of the observed GNSS satellites, and the absent ionospheric delays.

The multi-GNSS PPP processing strategies for sensing ZTDs in real time are detailed in Table 1. With the calculated ZHDs and estimated ZWDs, we can reconstruct the ZTDs as given below:

10 $${{\rm{Zt}}}_{r}={{\rm{Zh}}}_{r}+{{\rm{Zw}}}_{r}+\phi $$

where Zt_*r*_ denotes the reconstructed ZTDs, namely the PPP-derived ZTDs, which can be adopted to evaluate the multi-GNSS PPP performance for atmospheric parameter measurement in real time, and *φ* denotes residual tropospheric delays.

**Table 1:** Processing strategies of multi-constellation integrated PPP for real-time ZTD retrieval.

Items	Strategies
Observation	Type: code and carrier phase Signal: E1/E5a (Galileo); B1/B2 (BDS); G1/G2 (GLONASS); L1/L2 (GPS)
Weight for observation	Elevation-dependent weight
Sampling interval	30 s
Elevation mask angle	7°
Estimator	Kalman filter
Satellite clock and orbit	Fixed using real-time precise products
Phase wind-up effect	Corrected
Relativistic effect	Applied
Station displacement	Ocean tide loading, pole tide, Solid Earth tide, IERS Convention 2010
Earth rotation parameter	Fixed
Phase center offset (satellite)	Corrected using IGS values for all satellite systems
Phase center offset (receiver)	GPS/GLONASS: corrected using IGS values BDS/Galileo: corrected using IGS-derived GPS values
Phase center variation (satellite)	GPS/GLONASS: corrected using IGS values BDS/Galileo: corrected using IGS values but neglected for partial satellites
Phase center variation (receiver)	GPS/GLONASS: corrected using IGS values BDS/Galileo: corrected using IGS-derived GPS values
Station coordinate	Fixed using SINEX solutions
Receiver clock offset	Estimated as white noise process
Ionospheric delay	IF combined model: forming IF combinations to remove first-order effects UC model: estimated as random-walk process
Phase ambiguity	Estimated as constant values for each continuous tracking arc
ISB and IFB	Estimated as random-walk process
Tropospheric delay	ZHD: corrected using Saastamoinen model based on GPT2 model ZWD: estimated as random-walk process Gradient: estimated as random-walk process Mapping function: GMF

## Datasets and products

### Multi-GNSS observations

**Figure 1:**
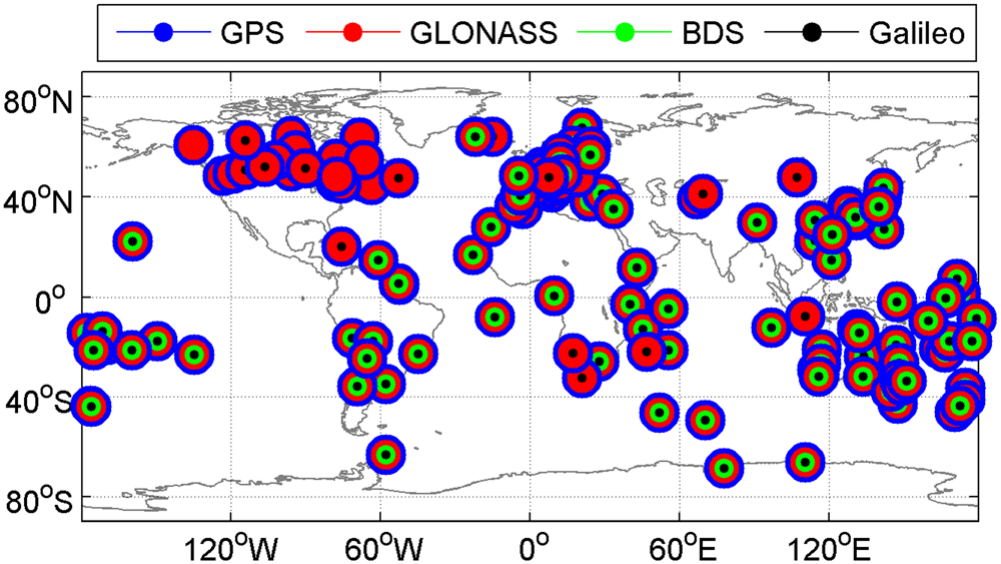
Geographical distribution of selected 160 MGEX stations and their respective tracking capability for different satellite systems. [The figure is plotted by MATLAB 2016a (https://cn.mathworks.com/products/matlab.html)].

For the purpose of incorporating the emerging and modernized satellite systems such as BDS and Galileo as well as modernized GPS and recovered GLONASS, the multi-GNSS Experiment (MGEX) was initiated by the IGS in 2012. The global MGEX ground tracking network has been deployed. Currently, there are more than 220 stations in the MGEX network and most of them allow for real-time data access besides the daily archival observations. We select 160 MGEX stations for analysis in this study, and their geographical distribution and tracking capability for different satellite systems are shown in Fig. 1. All selected stations are able to track the signals of GPS constellation, while the number of stations capable of tracking Galileo, BDS and GLONASS signals is 146, 120 and 158, respectively. A total of 120 stations support all four GNSS constellations. The real-time streams of observations from the 160 MGEX stations are received through Networked Transport of RTCM via Internet Protocol (NTRIP) and decoded in real time for ZTD retrieval with PPP. The real-time data are received from BKG (Bundesamt für Kartographie und Geodäsie) NTRIP Caster via the internet without any special licenses, while the only requirement is a user registration. More detailed information about the real-time data can be found on the BKG website (https://igs.bkg.bund.de). The datasets on April 2–8, 2017 are employed. In real-time processing, the workload of estimating ZTD in real time along with receiving real-time data streams from a global reference network is very heavy. Consequently, the analysis of real-time ZTD retrieval is confined to a short period of time. A week of datasets are enough to identify the performance of real-time PPP-derived ZTDs. Thus, we arbitrarily choose seven days to illustrate the obtained results. Actually, we have tested more datasets with a longer time span, and no significant performance differences for the real-time ZTD estimates could be found.

### Real-time precise satellite orbit and clock products

The IGS Real Time Working Group (RTWG) has officially provided the Real-Time Service (RTS) since 2013, which facilitates the real-time precise applications of GNSS. Currently, several analysis centers can provide the RTS products, including real-time precise corrections of satellite orbits and clocks. The RTS products can be received through NTRIP in real time. Most analysis centers only provide GPS or GPS/GLONASS RTS products, while all four GNSS systems can be supported by the RTS products from the analysis center CNES (Centre National d’Études Spatiales, France). Therefore, the CNES RTS products are adopted for analysis.

**Figure 2:**
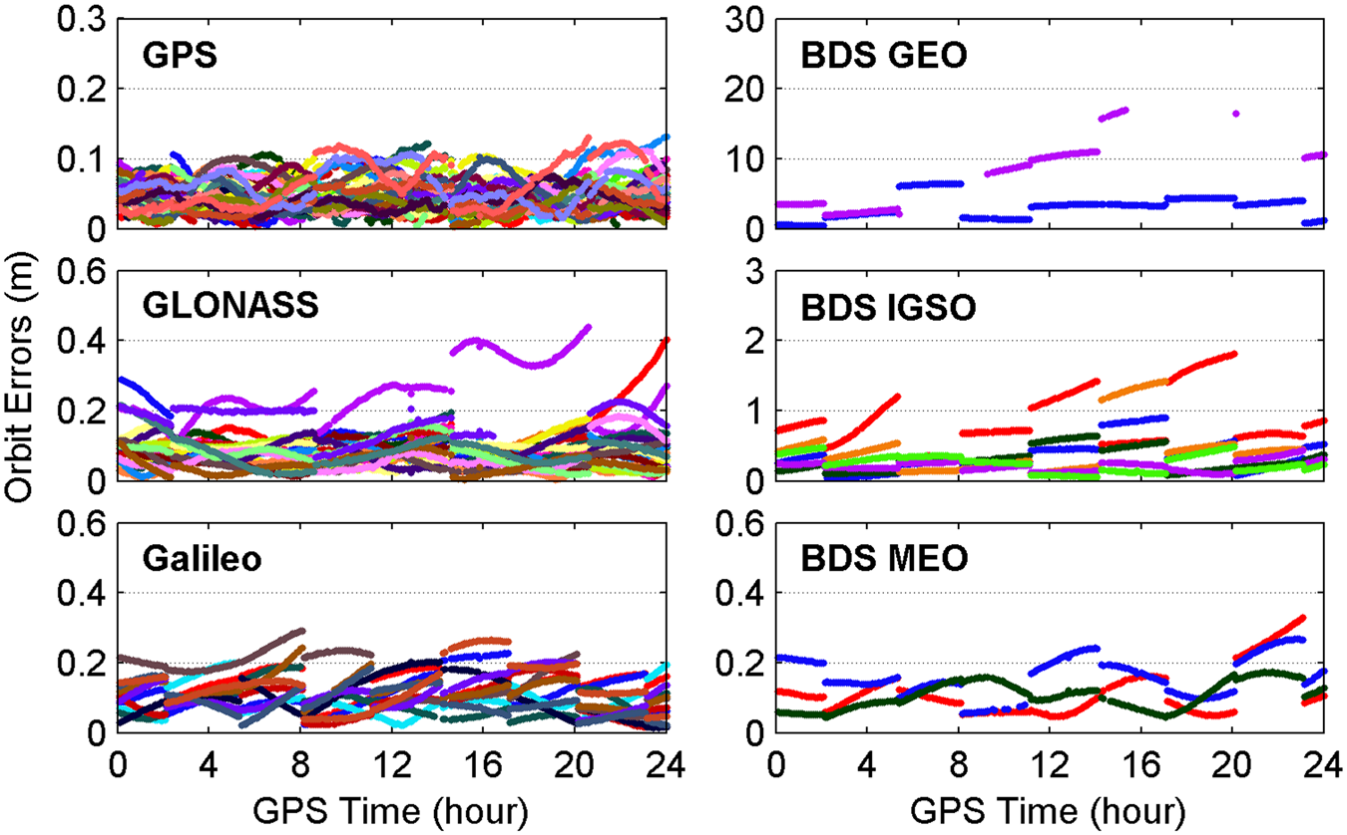
Errors of real-time satellite orbit corrections provided by CNES for different GNSS constellations on April 3, 2017. [The figure is plotted by MATLAB 2016a (https://cn.mathworks.com/products/matlab.html)].

**Figure 3:**
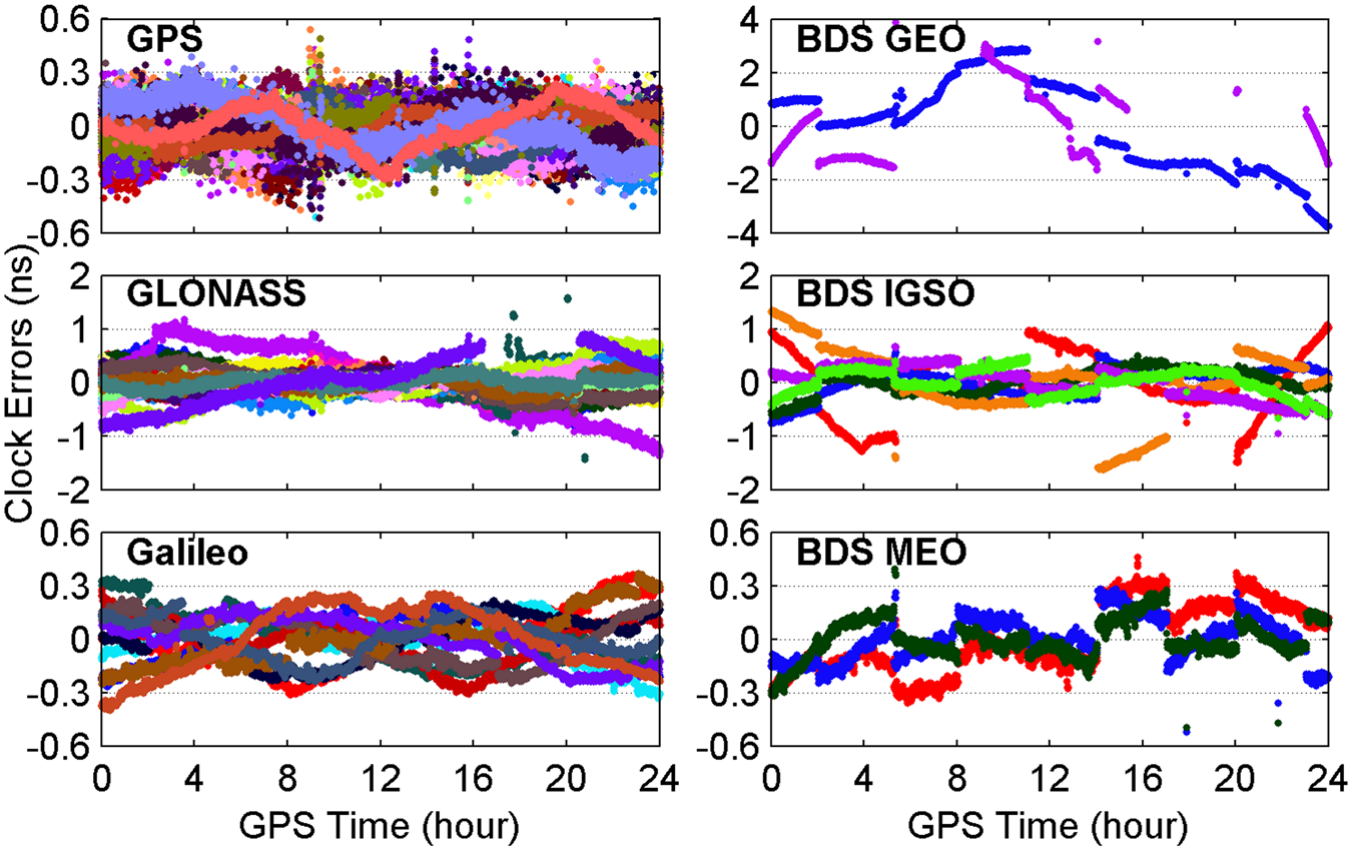
Errors of real-time satellite clock corrections provided by CNES for different GNSS constellations on April 3, 2017. [The figure is plotted by MATLAB 2016a (https://cn.mathworks.com/products/matlab.html)].

Since the accuracy and reliability of PPP-derived ZTDs depend on the performance of used RTS products, the quality of real-time satellite orbit and clock products provided by CNES is assessed by taking the GFZ (Deutsches GeoForschungsZentrum, Germany) final products as references. The errors of real-time satellite orbit and clock corrections provided by CNES for different GNSS constellations on April 3, 2017 are shown in Figs 2 and 3, respectively. The orbit three-dimensional (3D) errors of GPS satellites vary within a range of 0.1 m, while the corresponding varying range for GLONASS, Galileo and BDS Medium Earth Orbit (MEO) satellites is almost twice larger than that for GPS satellites. The orbit 3D errors are very large for BDS Inclined Geosynchronous Orbit (IGSO) and Geostationary Earth Orbit (GEO) satellites, and the corresponding varying range is 1 and 10 m, respectively. The GPS, Galileo and BDS MEO satellite clock errors all range from −0.3 to 0.3 ns, whereas the corresponding clock errors vary within a range of −1 to 1 ns for GLONASS and BDS IGSO satellites and of −4 to 4 ns for BDS GEO satellites.

For further analysis, Table 2 provides the root mean squares (RMSs) of errors of satellite orbit and clock corrections from CNES RTS products over April 2–8, 2017. The RMS values of clock and orbit errors for GPS satellites are 0.10 ns and 0.054 m, respectively, while the corresponding RMSs of GLONASS, Galileo and BDS MEO satellites are twice or even thrice larger than those of GPS satellites. The RMS statistics of BDS GEO and IGSO satellite orbit errors are increased to 7.322 and 0.596 m, respectively, while the accuracy of satellite clocks degrades to 2.08 and 0.54 ns, respectively.

**Table 2:** RMS values of errors of satellite orbit and clock corrections from CNES RTS products over April 2–8, 2017.

Constellation	Orbit (m)	Clock (ns)
GPS	0.054	0.10
GLONASS	0.122	0.25
Galileo	0.136	0.16
BDS GEO	7.322	2.08
BDS IGSO	0.596	0.54
BDS MEO	0.166	0.29

According to above accuracy analysis about real-time precise corrections of satellite orbits and clocks, the stochastic model of observations is determined. The precision of GPS code and phase observations is set to 0.3 and 0.003 m, respectively. The phase observation precision is set to 0.006 m and code observation precision is set to 0.6 m for GLONASS, Galileo and BDS MEO satellites since their real-time satellite orbits and clocks are at the relatively lower accuracy. The observations from BDS GEO and IGSO satellites are down-weighted with a factor of 2500 and 25, respectively, so as to mitigate the effects of much larger satellite orbit and clock errors.

### References of tropospheric ZTD estimates

The final tropospheric ZTD products provided by USNO (United States Naval Observatory, USA) and CODE (Center for Orbit Determination in Europe, Switzerland) are used as references to evaluate the performance of real-time ZTDs estimated with the four-constellation integrated PPP. Table 3 lists the information about the two final ZTD products. The accuracy of the CODE and USNO final ZTD products is at a level of 4 mm, with respect to the tropospheric products generated by other measurement techniques such as VLBI, DORIS and radiosondes^14,26^.

**Table 3:** Information about the final tropospheric ZTD products.

Items	CODE ZTD Products	USNO ZTD Products
Elevation mask angle	3°	7°
Sampling interval	3 min	5 min
Sampling ZTD	2 h	5 min
Solutions	Network solution	Network solution
Num. of stations	about 250 stations	about 350 stations
Processing	post-processing	post-processing
Software	Bernese GNSS Software (Version 5.3)	Bernese GPS Software (Version 5.0)
Mapping functions	Vienna Mapping Function	Global Mapping Function

## Results

### Real-time ZTDs derived from IF combined PPP

The real-time ZTDs are retrieved based on IF combined multi-GNSS PPP with the use of the CNES real-time satellite orbit and clock corrections. The limitation of PPP technique is that the reliable solutions of estimated parameters can only be obtained after conducting the PPP processing for a period of time, which is defined as convergence time. In this study, the ZTD estimates are considered to have converged when the ZTD errors are smaller than 20 mm for 10 consecutive epochs. The convergence time refers to the time span from the first epoch to the converged epoch. It is important to notice that the convergence time of the estimated ZTDs and the processing time of the employed equipment are two completely different concepts. The correlation between coefficient matrices of adjacent epochs is very high in the process of parameter estimation. Therefore, the repeat observation in a very short period of time will do little to help the convergence or the accuracy improvement of unknown parameters including the ZTDs because of the small changes in satellite sky distribution. In contrast, a longer observation time (at least several minutes for ZTD retrieval) can be very helpful, which benefits from the availability of numerous measurements with distinct coefficient matrices and the strong satellite geometry. The processing time for the observations at a single epoch from one station is usually tens of milliseconds. When simultaneously processing the datasets from hundreds of stations in real-time scenarios, the processing time for a single epoch is usually several seconds. Although we can reduce the processing time by adopting the better operating system (OS) platform and parallel computation so that the sampling interval can be reduced to receive much more measurements within a specific period of time, the convergence time of the ZTD estimates will not show significant differences. However, the higher sampling rate will improve the statistical accuracy of convergence time. For example, the convergence time may be 570 s when using the observations with a sampling interval of 30 s, while the corresponding convergence time may be 565 s when the observations are recorded at a sampling rate of 5 s. To validate our opinion, we have compared the post-processed ZTD results under the Linux OS and Windows OS. The results indicate that the convergence time of the ZTD estimates using the Linux OS is the same as that using the Windows OS, while the former OS has the shorter processing time. All the following real-time and post-processed ZTD results are derived from the Windows OS.

**Figure 4:**
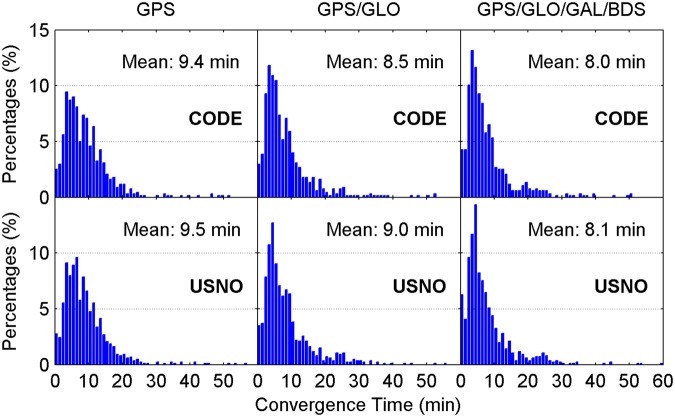
Distribution of convergence time of real-time ZTD estimates for different constellation combinations using all the 24-hour datasets from 160 MGEX stations spanning a week. The abbreviations GAL and GLO refer to Galileo and GLONASS, respectively. [The figure is plotted by MATLAB 2016a (https://cn.mathworks.com/products/matlab.html)].

**Figure 5:**
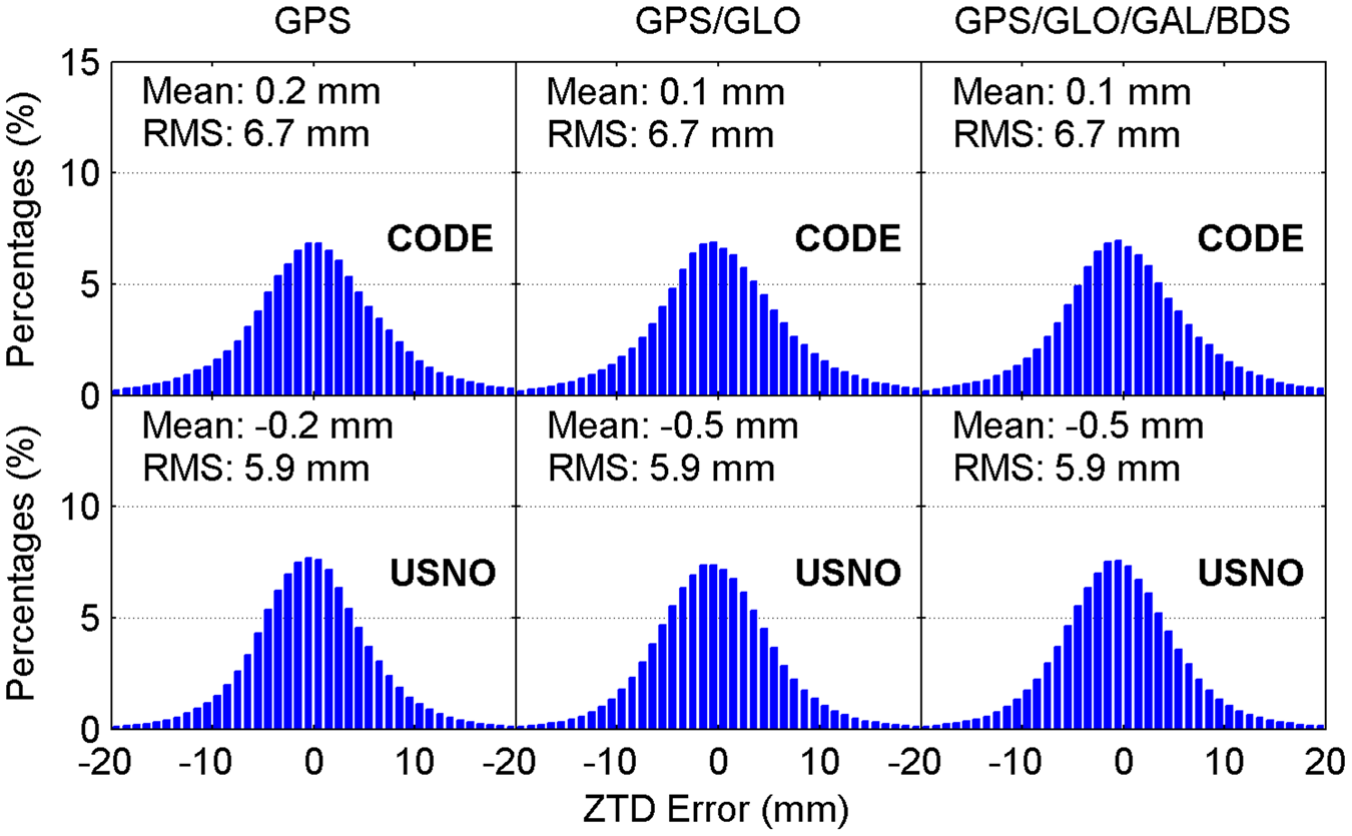
Distribution of epoch-wise errors of real-time ZTD estimates for different constellation combinations using all the 24-hour datasets from 160 MGEX stations spanning a week. [The figure is plotted by MATLAB 2016a (https://cn.mathworks.com/products/matlab.html)].

Figure 4 illustrates the distribution of convergence time of real-time ZTD estimates for different constellation combinations using all the 24-hour datasets from 160 MGEX stations spanning a week. It is clear that the percentages of convergence time shorter than 10 minutes increase as more satellite systems are involved in the processing. The average convergence time for all 24-hour sessions is also given in each panel. According to the average values, the improvement of real-time ZTDs on convergence time is 10% for the GPS/GLONASS case over the GPS-only case, and 6% for the four-constellation case over the GPS/GLONASS case with respect to the CODE final ZTD products, respectively. In addition, the corresponding convergence time improvement is 5% and 10% when taking the USNO final ZTD products as references, respectively. Figure 5 depicts the distribution of the epoch-wise ZTD errors. It is important to notice that the ZTD estimates in the converging stage are excluded from the accuracy statistics. The ZTD errors are approximately normally distributed for GPS-only, GPS/GLONASS and four-constellation cases. After the real-time ZTD estimates converge to the stable values, their accuracy is found to be at a same level for all three different constellation combination cases. The RMS statistics and average values of ZTD errors over all the available epochs are also shown in each sub-figure. Based on RMSs, the retrieval accuracy of real-time ZTDs is 5.9 and 6.7 mm with respect to the USNO and CODE final ZTD products, respectively. The average ZTD errors for all selected stations and days are –0.5 to –0.2 mm and 0.1 to 0.2 mm by comparing with the two final ZTD products, respectively.

**Figure 6:**
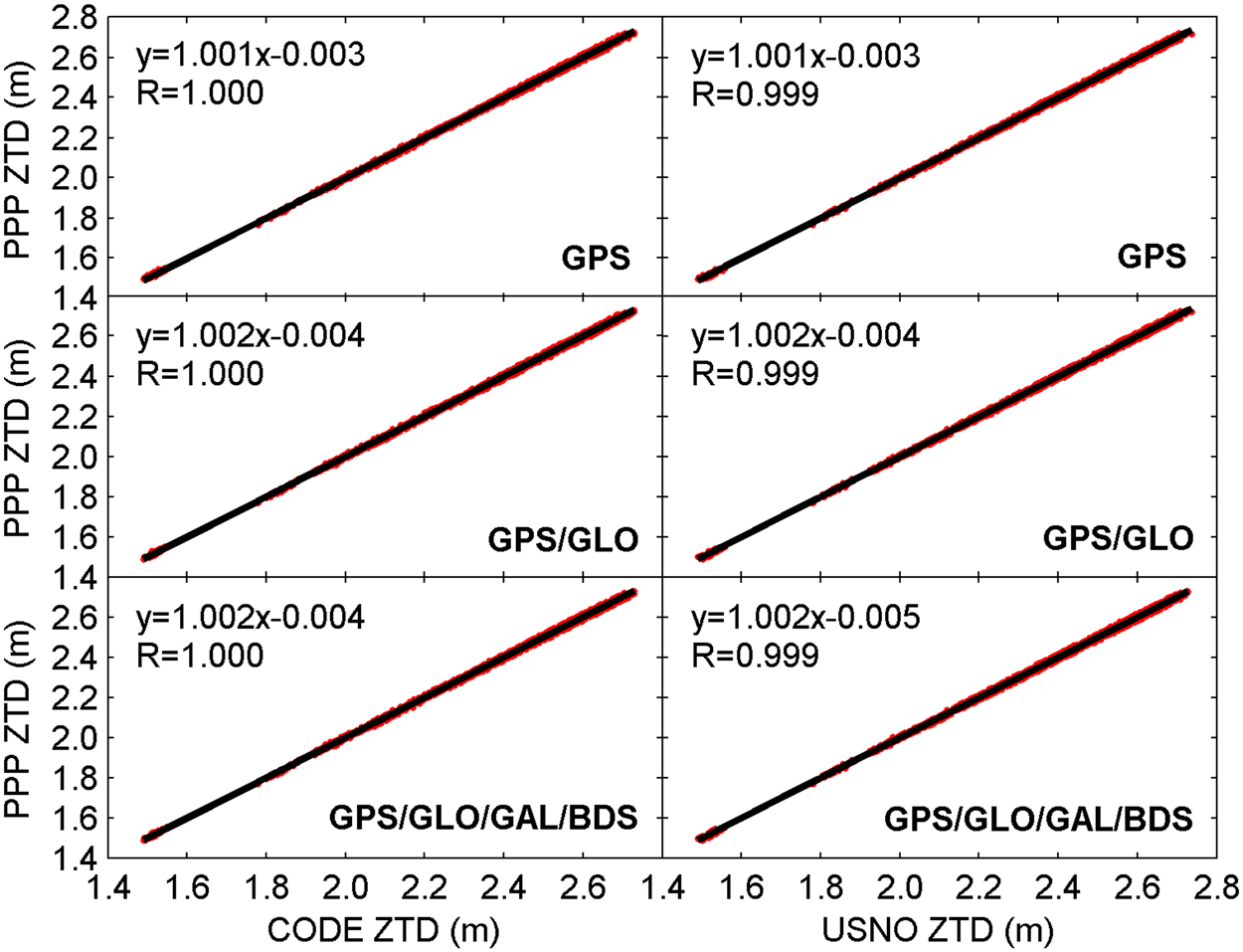
Linear fitting of real-time ZTDs derived from PPP to post-processed ZTDs provided by CODE and USNO. The correlation coefficient R and linear regression are also provided. [The figure is plotted by MATLAB 2016a (https://cn.mathworks.com/products/matlab.html)].

Figure 6 shows the linear fitting of real-time ZTDs derived from PPP to post-processed ZTDs provided by CODE and USNO. The correlation coefficient, marked as R, and linear regression are also provided in each panel. For either single constellation case or multi-GNSS integration case, the correlation coefficient between real-time ZTDs and post-processed ZTDs from CODE and USNO is 1.000 and 0.999, respectively, suggesting the strong correlation between real-time PPP-derived ZTDs and referenced ZTDs.

**Figure 7:**
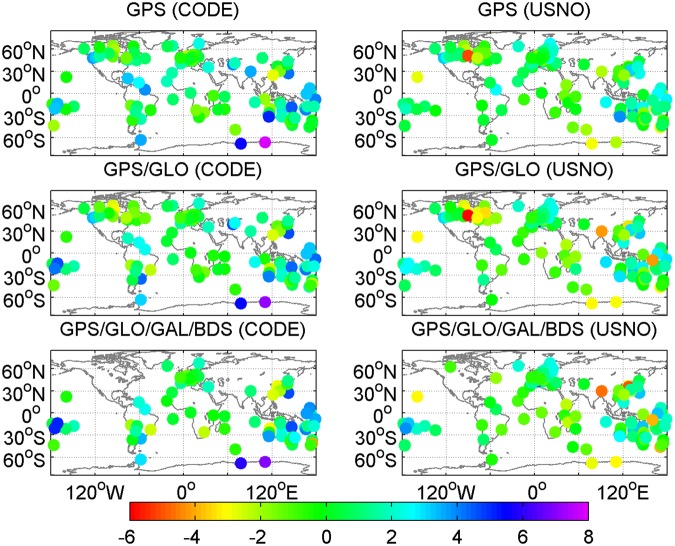
Geographical distribution of mean values of real-time ZTD errors at each station on April 2–8, 2017 (unit: mm). [The figure is plotted by MATLAB 2016a (https://cn.mathworks.com/products/matlab.html)].

**Figure 8:**
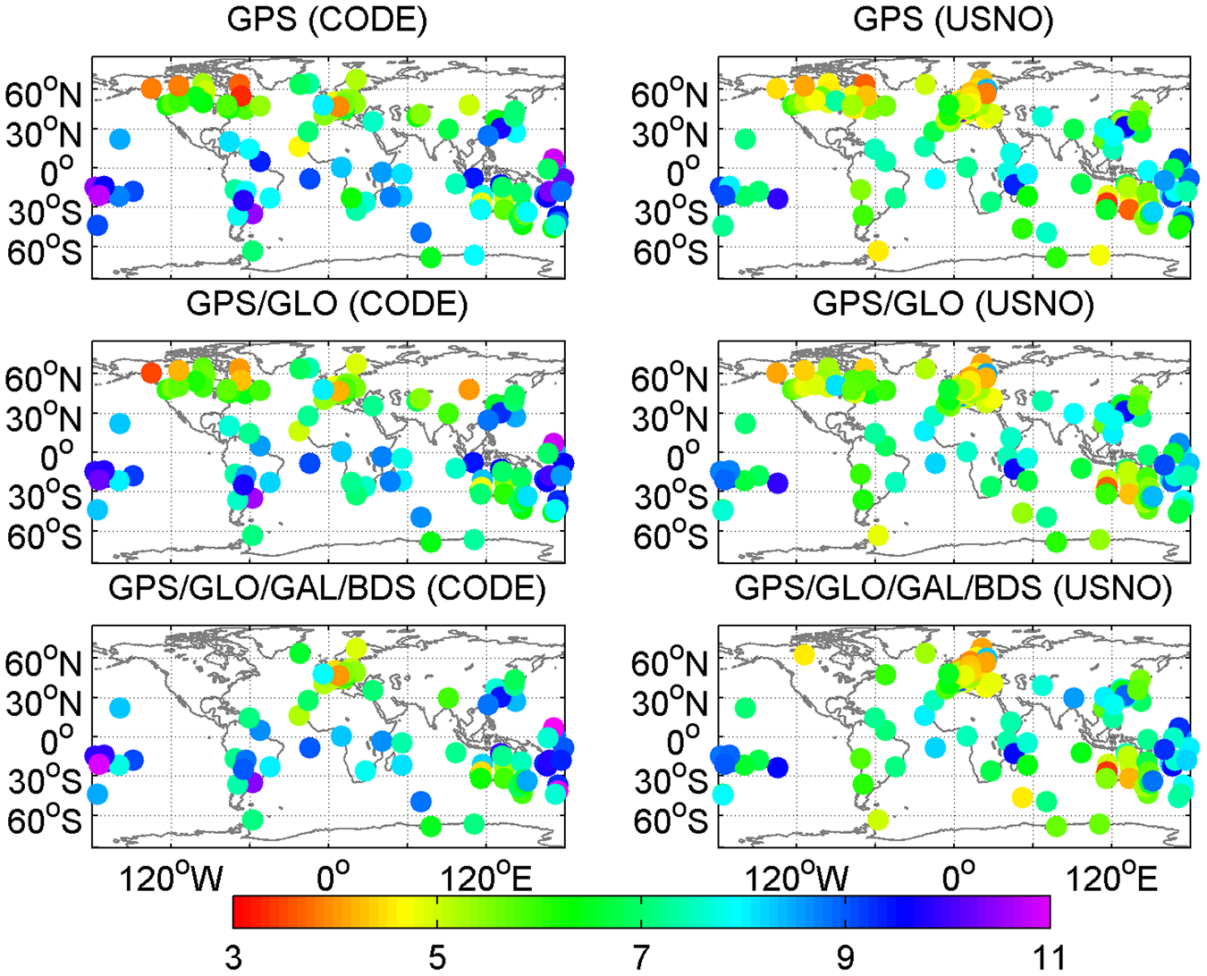
Geographical distribution of RMS values of real-time ZTD errors at each station on April 2–8, 2017 (unit: mm). [The figure is plotted by MATLAB 2016a (https://cn.mathworks.com/products/matlab.html)].

**Figure 9:**
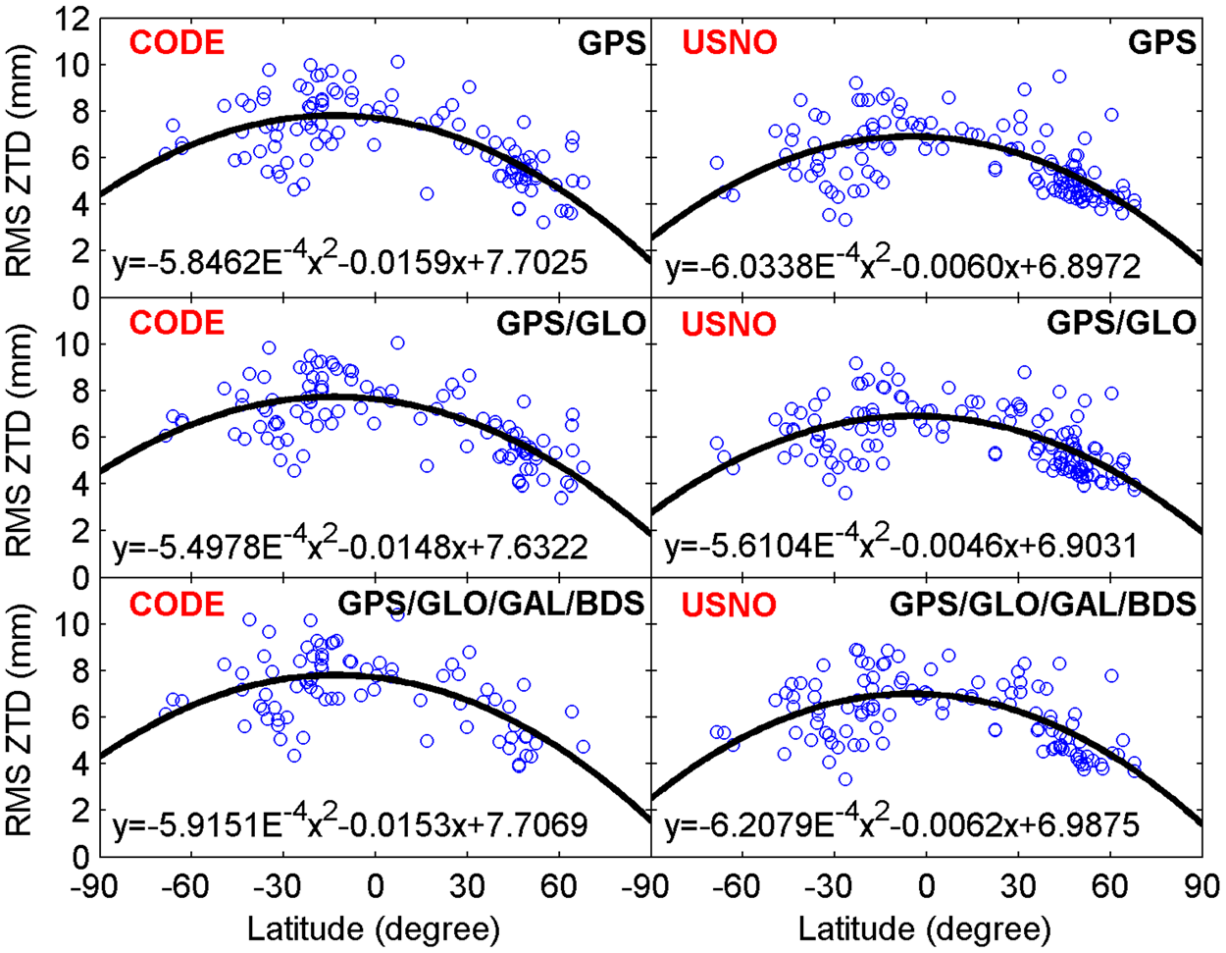
Station-specific RMS values of real-time ZTD errors as a function of geographical latitudes. The black line refers to the second-order polynomial fitting of RMS ZTD errors. [The figure is plotted by MATLAB 2016a (https://cn.mathworks.com/products/matlab.html)].

Figures 7 and 8 provide the geographical distribution of station-specific statistics of real-time ZTD errors in terms of mean values and RMS values, respectively. The average ZTD errors at a station range from −6 to 8 mm, while the station-specific RMS ZTD errors vary within a range of 3 to 11 mm. The results indicate that there is a good agreement between post-processed ZTDs and real-time ZTDs. In addition, the station-specific RMS ZTD errors for low-latitude stations are larger than those for high-latitude stations. Therefore, the station-specific RMS ZTD errors depend on the geographical latitudes. For further analysis, the station-specific RMS values of real-time ZTD errors are expressed as a function of latitudes, as shown in Fig. 9. The black line refers to the second-order polynomial fitting of RMS ZTD errors, and reveals a trend that the retrieval accuracy of real-time ZTDs increases as the geographical latitude increases. This phenomenon can be attributed to the different atmospheric water vapor contents in different latitude regions. The PWV contents are lower in dry (high-latitude) regions, and higher in moist (low-latitude) regions. The estimation accuracy of ZWD/PWV is confined to its fast spatiotemporal variability.

For further analysis, a longer analysis period from June 1 to 15, 2017 is adopted. The datasets from the stations shown in Fig. 1 during this period of time are processed. The obtained results are similar to those shown in Figs 6 and 9, which further verifies the strong correlation between real-time PPP-derived ZTDs and post-processed ZTDs, as well as the dependence of real-time ZTD retrieval accuracy on geographical latitude.

### Effects of tropospheric asymmetry on real-time ZTD retrieval

In order to investigate the effects of tropospheric asymmetry on real-time ZTD retrieval, the four-constellation integrated real-time PPP processing with and without consideration of the tropospheric horizontal gradients in IF combined model is carried out. After considering the tropospheric gradients, the retrieval accuracy of real-time ZTDs is slightly improved from 7.89 to 7.33 mm in comparison to the case ignoring the two horizontal gradients. The epochs in both converging and converged stages for all selected stations and days are involved in the accuracy statistics, and the USNO final ZTD products are used as references. Although the accuracy improvement is marginal, it is better not to neglect the effects of tropospheric asymmetry because of the pursuit of high-accuracy ZTDs.

The above ZTD results are obtained, provided that the tropospheric gradients are estimated as random-walk process. In some studies, the tropospheric gradients were modeled as piecewise constants so that the effects of gradient estimation with different temporal resolutions on the position estimates could be analyzed^21,27^. For completeness, the effects of the used dynamic models of the tropospheric gradient parameters in the Kalman filter on the ZTD calculation are also investigated in this study. For comparison, the tropospheric gradients are estimated with five different intervals, namely 12, 6, 4, 2 and 1 h. Table 4 provides the retrieval accuracy of real-time ZTDs with the use of different temporal resolutions for the estimation of tropospheric horizontal gradients. Compared with the ZTD solutions ignoring tropospheric azimuthal asymmetry, the retrieval accuracy of the ZTD solutions considering the horizontal gradients in an interval of 12 h is improved by 4% from 7.89 to 7.61 mm. The ZTD retrieval accuracy is continuously improved with increasing temporal resolutions for the estimation of horizontal gradients. When the temporal resolution is set to 1 h, the ZTD retrieval accuracy is comparable to that of the solutions modeling the tropospheric gradients as random-walk process. Therefore, the gradient estimation with high temporal resolution should be performed for the real-time retrieval of ZTDs. It is important to note that the tropospheric asymmetry becomes much significant in the vicinity of severe weather phenomena. In this situation, the random-walk process rather than the piecewise constants should be employed for the gradient parameters.

**Table 4:** Retrieval accuracy of real-time ZTDs with the use of different temporal resolutions for the estimation of tropospheric horizontal gradients.

Temporal Resolution	ZTD Retrieval Accuracy (mm)
12 h	7.61
6 h	7.48
4 h	7.39
2 h	7.35
1 h	7.33

### Real-time ZTDs in constrained visibility environments

**Figure 10:**
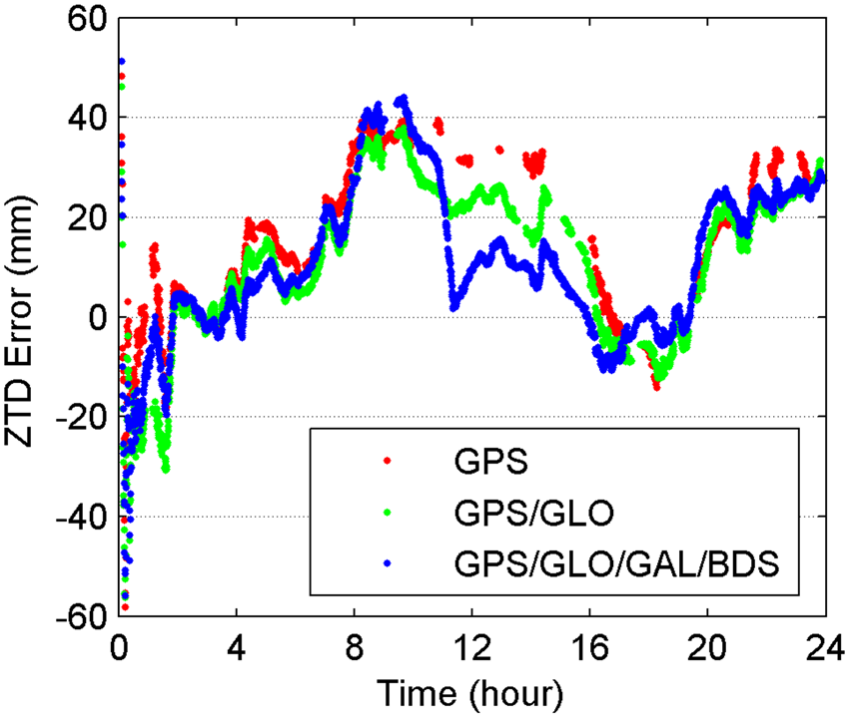
Errors of real-time ZTD estimates with a satellite elevation mask angle of 40° at station CEDU on April 8, 2017. [The figure is plotted by MATLAB 2016a (http://cn.mathworks.com/products/matlab.html)].

Very often we need to obtain the atmospheric parameters in the constrained visibility environments, such as open-pit mines, mountainous areas and urban canyons. To simulate these real harsh environments, the elevation mask angles are increased from 10° to 40° in steps of 10°. The performance of multi-GNSS PPP for real-time retrieval of ZTDs in environments with limited satellite visibility is investigated. Figure 10 shows the errors of real-time ZTD estimates with a satellite elevation mask angle of 40° at station CEDU on April 8, 2017 for GPS-only, GPS/GLONASS and four-constellation cases. The final ZTD products provided by USNO are taken as reference datasets. It is clear that the real-time ZTDs cannot be retrieved at many epochs for GPS-only PPP, especially over the period of 9:56–16:02. In addition, the joint use of multi-GNSS signals significantly reduces the ZTD errors during this period of time. At a cut-off elevation angle of 40°, the real-time ZTD errors vary within a range of −40 to 40 mm. Table 5 provides the average availability and RMS statistics of errors of real-time ZTD estimates for 10 stations on April 8, 2017. The 10 stations are chosen because they are able to track four-constellation signals and located in Asia-Pacific area covered by BDS service. The availability is defined as the percentage of the epochs with capability of retrieving real-time ZTDs over the total epochs during a day. The availability decreases with increasing cut-off elevation angles. The availability is reduced from 100.0% to 95.5% for the GPS-only case when the elevation mask angle is increased from 10° to 30°, whereas the corresponding availability is always larger than 99.9% for the dual- and four-constellation cases. At a mask angle of 40°, the availability drops to 85.9% and 45.1% for the GPS/GLONASS and GPS-only cases, respectively. On the contrary, the availability of the four-constellation case still remains 95.3%. As to the measurement accuracy of real-time ZTDs with different cut-off elevation angles, it continuously degrades along with the increment of cut-off elevation angles. After the integration with more GNSS systems, the ZTD retrieval accuracy is only slightly improved, even for a cut-off angle of 40°. The reason for the small improvement is that more epochs with larger ZTD errors are involved in the RMS statistics for the multi-constellation cases. The RMS values of real-time ZTD errors over all the common epochs are 18.76, 17.70 and 17.06 mm for single-, dual- and four-constellation cases at a mask angle of 40°, respectively. In addition, the relatively lower accuracy level of real-time precise corrections for GLONASS, Galileo and BDS satellite orbits and clocks limits the contribution of multi-GNSS combination.

**Table 5:** Average availability and RMS statistics of errors of real-time ZTD estimates for 10 stations on April 8, 2017.

Elevation Mask Angle (degree)	Constellation Combination	Availability (%)	ZTD Error (mm)
10°	GPS	100.0	7.13
	GPS/GLO	100.0	7.02
	GPS/GLO/GAL/BDS	100.0	6.92
20°	GPS	99.8	8.87
	GPS/GLO	100.0	8.81
	GPS/GLO/GAL/BDS	100.0	8.12
30°	GPS	95.5	12.69
	GPS/GLO	99.9	12.59
	GPS/GLO/GAL/BDS	99.9	12.37
40°	GPS	45.1	19.23
	GPS/GLO	85.9	19.03
	GPS/GLO/GAL/BDS	95.3	18.90

As shown in Table 5, the retrieval accuracy of real-time ZTDs significantly degrades with the increment of elevation mask angles, even for the four-constellation case, but the four-constellation case still achieves the highest ZTD estimation accuracy and the multi-constellation combination can help to dramatically compensate the availability. It seems that the real-time ZTD retrieval will show good performance provided that the cut-off elevation angle is set to 10°. However, the satellite signals with relatively low elevations (larger than 10°) will be blocked by the surroundings near the station when the real-time ZTD retrieval is carried out in the harsh environments such as the urban canyons and open-pit mines, in spite of the elevation mask angle of 10°. In this case, only the observations with relatively high elevations (larger than 20°, 30° or 40° based on the specific conditions) can be involved in the processing. In this study, we simply simulate the real harsh environments by setting different high cut-off elevation angles so that the performance of real-time ZTD estimates in these environments can be evaluated.

### Real-time ZTDs derived from UC PPP

**Figure 11:**
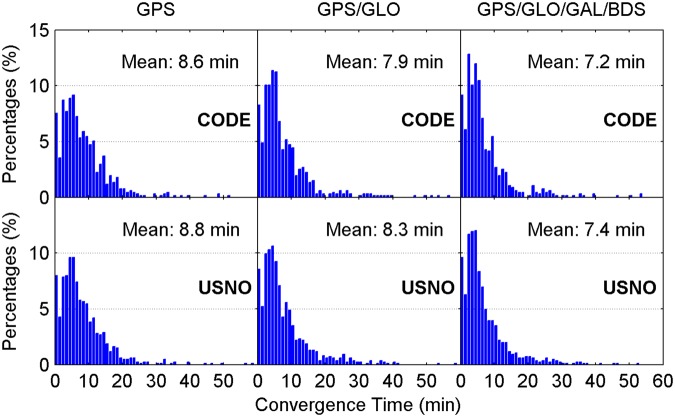
Distribution of convergence time of real-time ZTD estimates derived from UC PPP. [The figure is plotted by MATLAB 2016a (https://cn.mathworks.com/products/matlab.html)].

**Figure 12:**
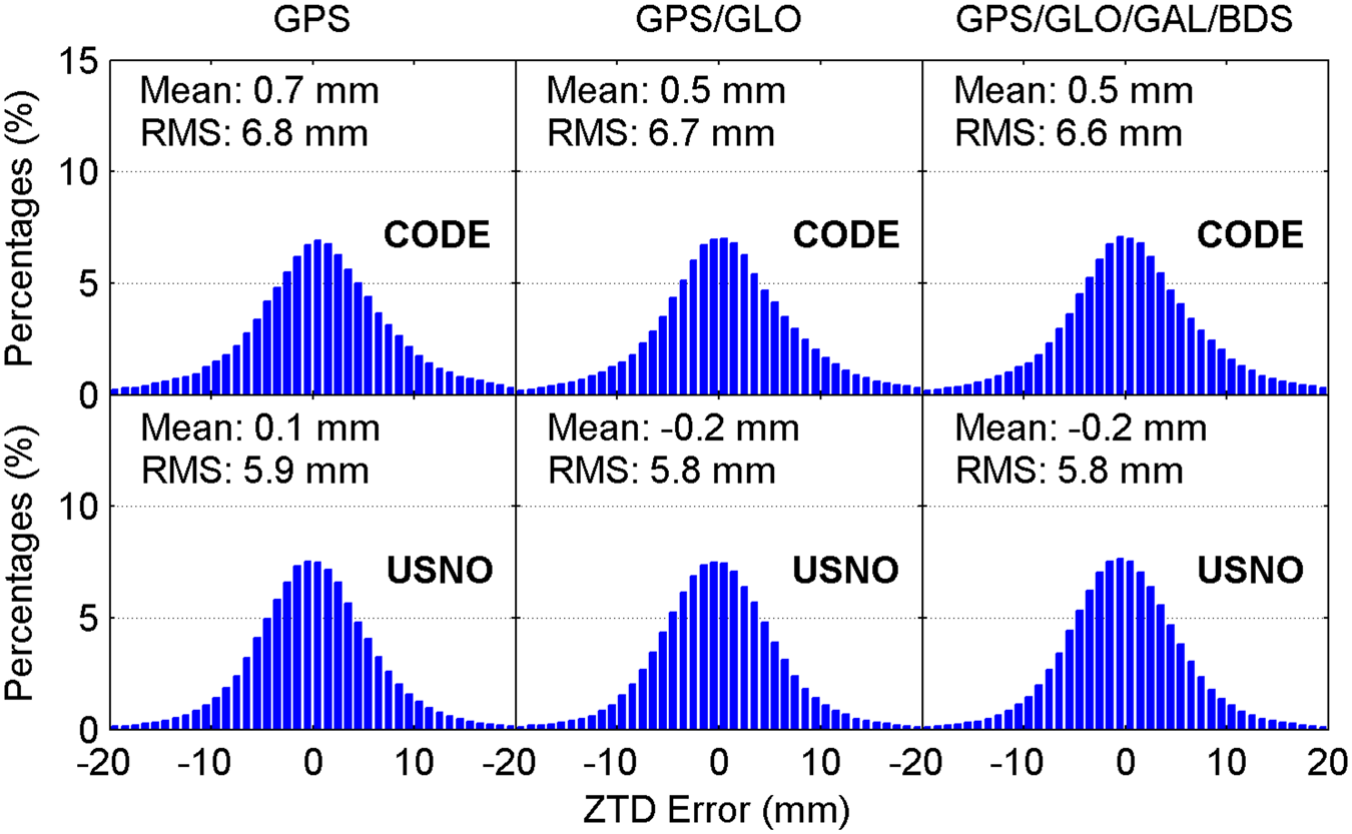
Distribution of epoch-wise errors of real-time ZTD estimates derived from UC PPP. [The figure is plotted by MATLAB 2016a (https://cn.mathworks.com/products/matlab.html)].

For comparison, the multi-constellation integrated PPP based on UC observations is also used to retrieve the ZTDs in real time. Figures 11 and 12 show the distribution of convergence time and epoch-wise errors of real-time ZTD estimates derived from UC PPP, respectively. The GPS/GLONASS PPP reduces the convergence time of real-time ZTD estimates by 8% and 6% over the GPS-only PPP when taking the CODE and USNO final ZTD products as references, respectively, while the corresponding improvement of four-constellation integrated PPP on ZTD convergence time is 9% and 11% over the GPS/GLONASS PPP, respectively. The real-time PPP using the observations from a single constellation or multi-GNSS constellations can achieve comparable estimation accuracy of ZTDs after the respective ZTD estimates reach stable values. In conjunction with the ZTD results shown in Figs 4 and 5, it is concluded that the real-time ZTDs retrieved from the UC PPP converge more quickly than those derived from the IF combined PPP, and the improvement of convergence time is 7–10%. The reason for the convergence time improvement may be that the temporal correlation of ionospheric delays is carefully considered in the UC model. We also analyze the effects of tropospheric azimuthal asymmetry on ZTD estimation for the UC model. When ignoring the tropospheric horizontal gradients, the accuracy of real-time PPP-derived ZTDs with four constellations degrades to 6.2 mm with respect to the USNO final ZTD products.

### Post-processed ZTDs derived from IF combined PPP

**Figure 13:**
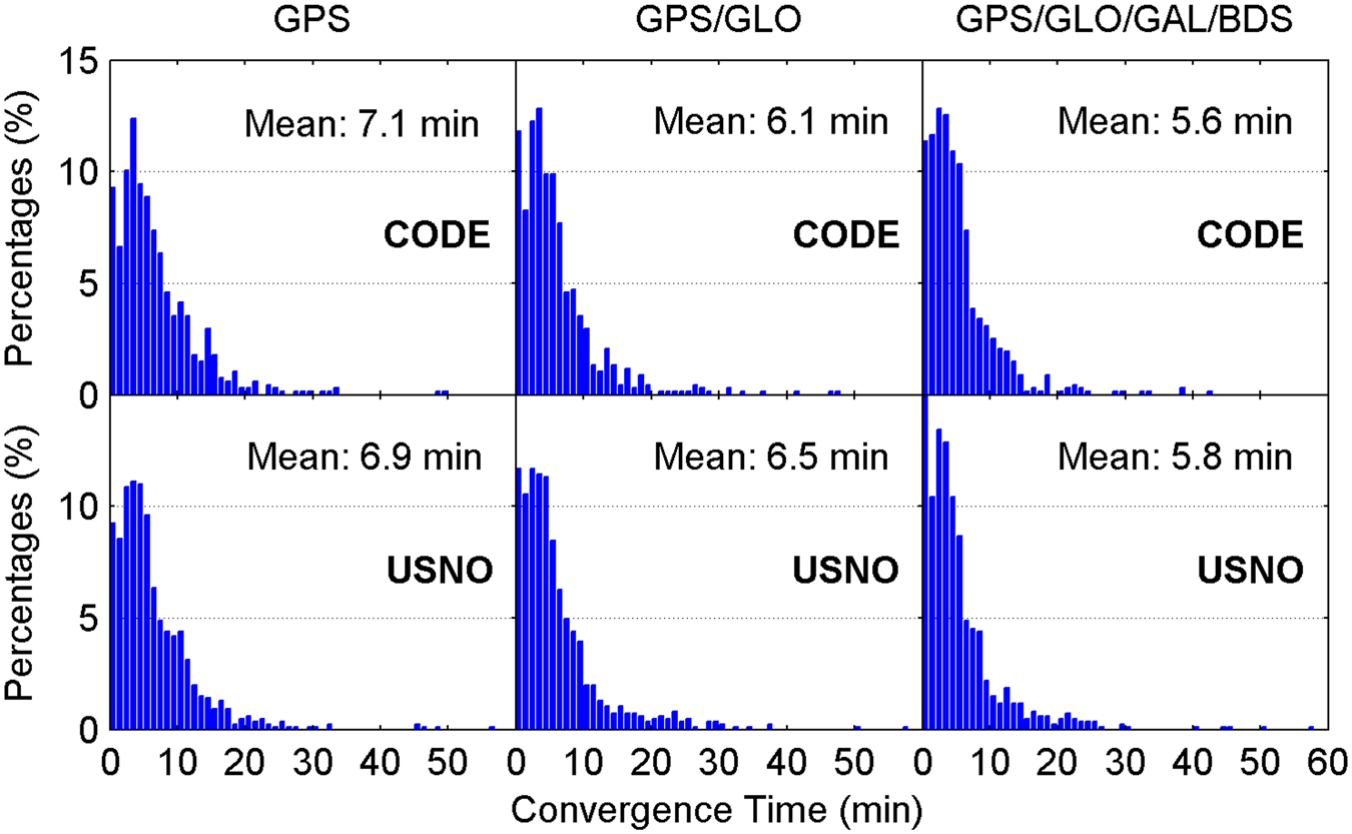
Distribution of convergence time of post-processed ZTD estimates derived from IF combined PPP. [The figure is plotted by MATLAB 2016a (https://cn.mathworks.com/products/matlab.html)].

**Figure 14:**
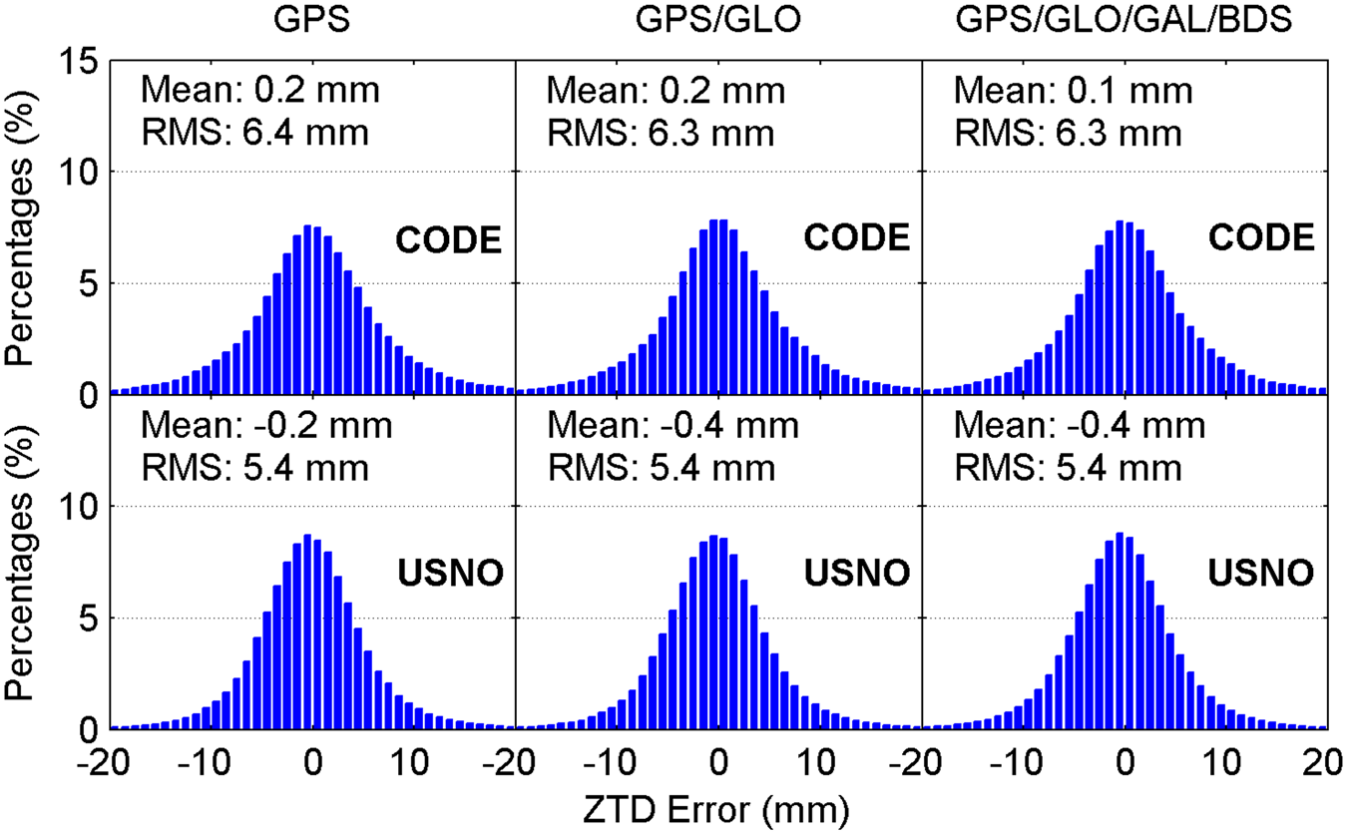
Distribution of epoch-wise errors of post-processed ZTD estimates derived from IF combined PPP. [The figure is plotted by MATLAB 2016a (https://cn.mathworks.com/products/matlab.html)].

For completeness, the post-processed ZTDs, namely the ZTDs estimated with multi-GNSS PPP using GFZ final satellite orbit and clock products, are analyzed. The IF combined model is adopted. The CODE and USNO final ZTD products derived from the network solutions are also taken as references. Figures 13 and 14 show the distribution of convergence time and epoch-wise errors of post-processed ZTD estimates for all selected days and stations, respectively. The GPS/GLONASS case improves the convergence time of post-processed ZTDs by 14% and 6% over the GPS-only case with respect to the CODE and USNO ZTD products, respectively. After further adding the Galileo and BDS observations to the PPP processing, the convergence time is further improved by 8% and 11% to 5.6 and 5.8 min with respect to the two final ZTD products, respectively. Similar to the results shown in Figs 5 and 12, the retrieval accuracy of post-processed ZTDs using PPP based on different constellation combinations is found to be at the same level when the results in the converging stage are removed. The measurement accuracy of post-processed ZTDs is 5.4–6.4 mm. Compared with the real-time ZTDs in IF combined model, the convergence time and estimation accuracy for the post-processed ZTDs have an improvement of 24–30% and 4–8%, respectively.

### Benefits of BDS constellation on ZTD calculation

**Figure 15:**
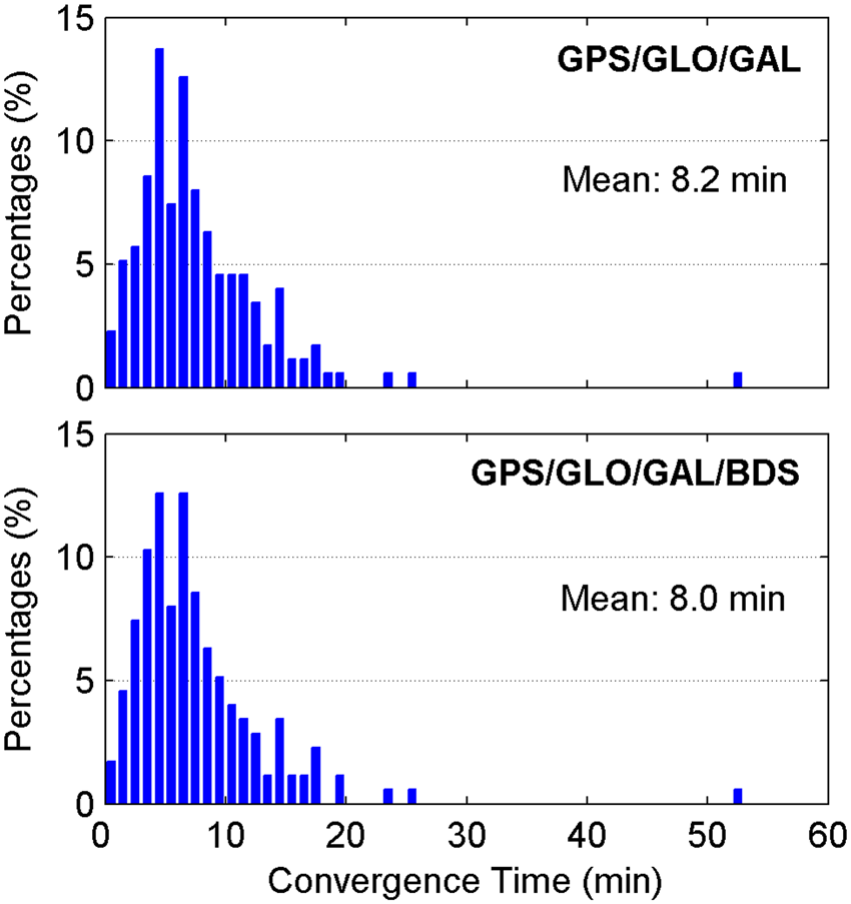
Distribution of convergence time of real-time ZTD estimates derived from IF combined PPP using the data at the stations located in Asia-Pacific area. [The figure is plotted by MATLAB 2016a (https://cn.mathworks.com/products/matlab.html)].

**Figure 16:**
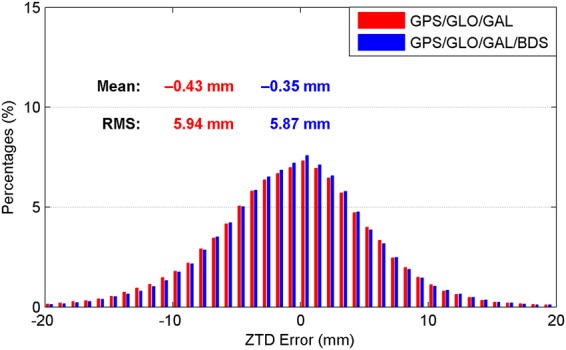
Distribution of epoch-wise errors of real-time ZTD estimates derived from IF combined PPP using the data at the stations located in Asia-Pacific area. [The figure is plotted by MATLAB 2016a (https://cn.mathworks.com/products/matlab.html)].

As shown in Table 2, the accuracy of real-time satellite orbit and clock corrections for BDS is significantly worse than that for the other three GNSS constellations. In spite of this, the BDS constellation is expected to benefit the ZTD retrieval, as long as the proper stochastic models are determined for the observations. To investigate the benefits from the BDS data on ZTD calculation, we compare the ZTD estimates derived from the real-time GPS/GLONASS/Galileo and GPS/GLONASS/Galileo/BDS combined PPP. Since the BDS mainly provides regional services at present, only the stations located in the Asia-Pacific area are selected for analysis. The datasets on April 2–8, 2017 are adopted. The IF combined model is employed. The USNO final ZTD products are used as references. Figures 15 and 16 show the obtained ZTD results in terms of convergence time and estimation accuracy, respectively. The real-time ZTD estimates that have not yet converged are not included in the accuracy statistics. After adding the BDS data, the convergence time is only shortened by 2%. As for the retrieval accuracy of real-time ZTDs, its improvement is also marginal. However, the percentages of the ZTD estimates with smaller errors increase after a further combination with BDS. The ZTD errors of smaller than 4 mm account for 27.90% for the triple-constellation case, while the corresponding percentages are increased to 28.73% for the four-constellation case. For further analysis, the performance of real-time ZTDs computed with BDS-only PPP is also evaluated. The convergence time and retrieval accuracy of real-time BDS-only PPP-derived ZTDs are 18.2 min and 9.6 mm, respectively, which are significant worse than those from GPS-only PPP shown in Figs 4 and 5. For the real-time ZTD calculation, greater benefits of BDS constellation can be anticipated with more available satellites and improved real-time precise corrections in the future.

## Conclusion and Discussion

The accuracy of four-constellation mixed real-time precise satellite orbit and clock products provided by CNES is assessed with respect to the GFZ final products. With the RTS products and multi-GNSS observations, the real-time PPP processing is conducted to retrieve the tropospheric ZTDs in real time. The final tropospheric ZTD products provided by CODE and USNO are taken as references. A week of datasets from 160 stations are employed.

The RMS values of clock errors and orbit 3D errors for GPS satellites are 0.10 ns and 0.054 m, respectively. Compared with GPS satellites, the corresponding RMSs of GLONASS, Galileo and BDS MEO satellites are increased by a factor of two to three times. The orbit accuracy degrades to 7.322 and 0.596 m for BDS GEO and IGSO satellites, respectively, while the corresponding clock accuracy is reduced to 2.08 and 0.54 ns, respectively.

The ZTDs retrieved from different constellation combinations, different processing models for ionospheric delays, and different modes are compared. The performance of retrieved ZTDs is evaluated in terms of convergence time and accuracy. The improvement of the GPS/GLONASS PPP on the convergence time is 5–14% over the GPS-only case. Compared with the dual-constellation case, the four-constellation case reduces the convergence time by 6–11%. The average convergence time is 8.0–8.1, 7.2–7.4, and 5.6–5.8 min for the four-constellation case in three different situations, namely IF combined PPP in real-time mode, UC PPP in real-time mode, and IF combined PPP in post-processing mode. If we only consider the ZTD estimates that have converged to the stable values, their retrieval accuracy is found to be at the same level for single-, dual- and four-constellation cases. When employing the three different situations, the retrieval accuracy of ZTDs is 5.9–6.7, 5.8–6.8, and 5.4–6.4 mm, respectively. The RMS values of real-time ZTD errors at a station are latitude dependent. With careful consideration of tropospheric gradients in the PPP processing, the accuracy of real-time ZTD estimates is slightly improved compared with the case ignoring atmospheric horizontal distribution. The multi-GNSS combination can also improve the availability of real-time PPP-derived ZTDs. The availability for the four-constellation case is improved from 45.1% to 95.3% over the GPS-only case at an elevation mask angle of 40°.

Compared with the previous studies, we improve the ZTD estimation due to the careful consideration of four aspects, namely the processing mode, data for ZTD retrieval, modeling of the tropospheric delays, and handing strategies of ionospheric delays. For the real-time PPP-derived ZTDs using GPS, GLONASS, Galileo and BDS data in the UC model, the convergence time and estimation accuracy can reach 7.4 min and 5.8 mm with respect to the USNO final ZTD products, respectively. Since the performance of these real-time ZTD estimates is only slightly worse than that of post-processed ZTD estimates, they can be used for the innovative applications, such as severe weather nowcasting. The convergence time can be increased to 8.8 and 8.3 min for the GPS-only and GPS/GLONASS real-time PPP-derived ZTDs, respectively. When neglecting the effects of tropospheric azimuthal asymmetry, the retrieval accuracy of real-time ZTDs degrades to 6.2 mm. As for the IF combined model, the convergence time can be lengthened to 8.1 min. Therefore, all the four aspects must be carefully taken into account during the ZTD estimation.

The improvement in the estimation of atmospheric parameters for our results can be attributed to the following several aspects. On the one hand, multi-constellation integrated PPP has the potential to significantly improve the retrieval performance of real-time ZTDs due to the increased number of visible satellites and the enhanced satellite sky distribution, especially when ZTD sensing is performed in areas with GNSS signal blockages. On the other hand, the highly variable atmospheric water vapor cannot be adequately modeled by a mapping function, assuming that the water vapor distribution has symmetry in all azimuth directions. Thus, the acquisition of enhanced ZTDs can benefit from the accurate estimation of tropospheric horizontal gradients. In addition, the UC model outperforms the IF combined model in terms of real-time ZTD retrieval because of the smaller observation noises as well as the careful consideration of the temporal correlation for the ionospheric delays. The above efforts are able to significantly improve the performance of real-time ZTD estimates, so that they can satisfy the requirements of GNSS meteorological application for nowcasting.

The accuracy of real-time precise satellite orbit and clock corrections for BDS satellites is much lower than that for GPS satellites. It is expected that the quality of RTS products will be improved due to the increasing ground tracking stations, especially for the BDS satellites. More new satellites will be launched in the future, which can benefit the satellite-based technology, such as PPP, as the Galileo and BDS are still in the stage of global deployment. With more available GNSS satellites and improved GNSS ephemerides, the real-time retrieved ZTDs with higher accuracy can be achieved by the real-time four-constellation integrated PPP technology.

## Data Availability

The data used to support the findings of this study are available from the corresponding author upon request.
